# Supplementary material for: Genome-Wide Identification of GATA Family Genes in Potato and Characterization of StGATA12 in Response to Salinity and Osmotic Stress
Source: Int J Mol Sci. 2024 Nov 19;25(22):12423. doi: 10.3390/ijms252212423 (PMC11594768; doi:10.3390/ijms252212423)
Supplement: Supplementary file 1 [file ijms-25-12423-s001.zip › Supplementary Materials.docx]

**Supplementary Figures**


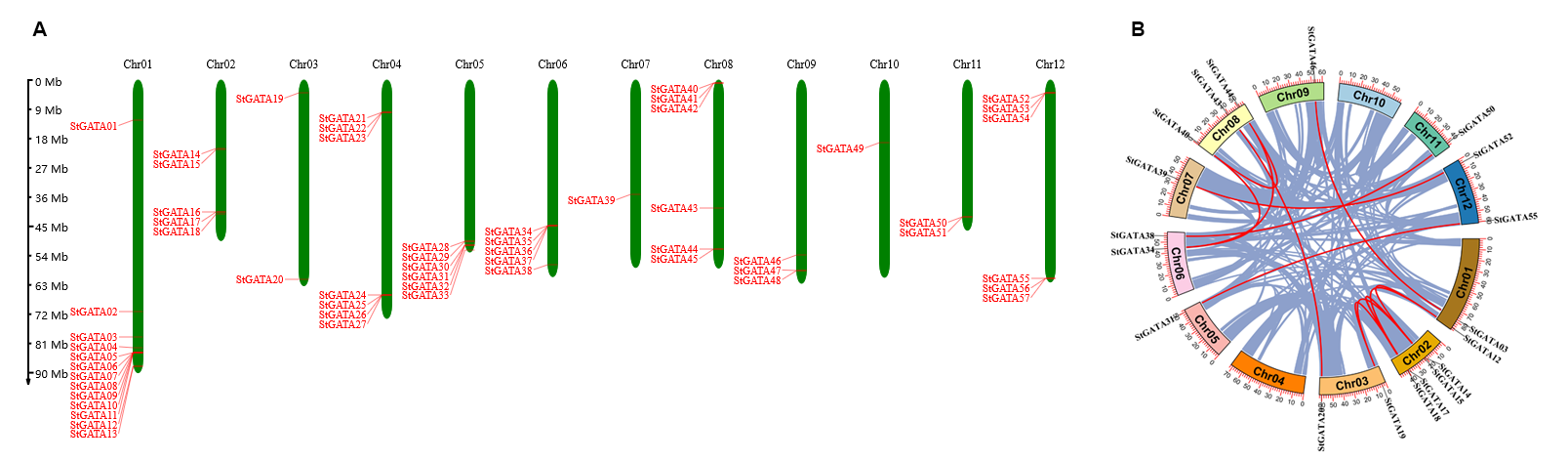


**Supplementary Figure S1.** Chromosomal localization, segmental duplication, and phylogenetic tree of StGATA proteins. (A) Chromosomal localization of *StGATA* genes in potato plants. (B) Schematic representations of inter-chromosomal relationships of *StGATAs*. Gene duplication events were analyzed by MCScanX. Blue lines inside present all synteny blocks in the potato genome. The segmentally duplicated genes were linked by the bold red line.


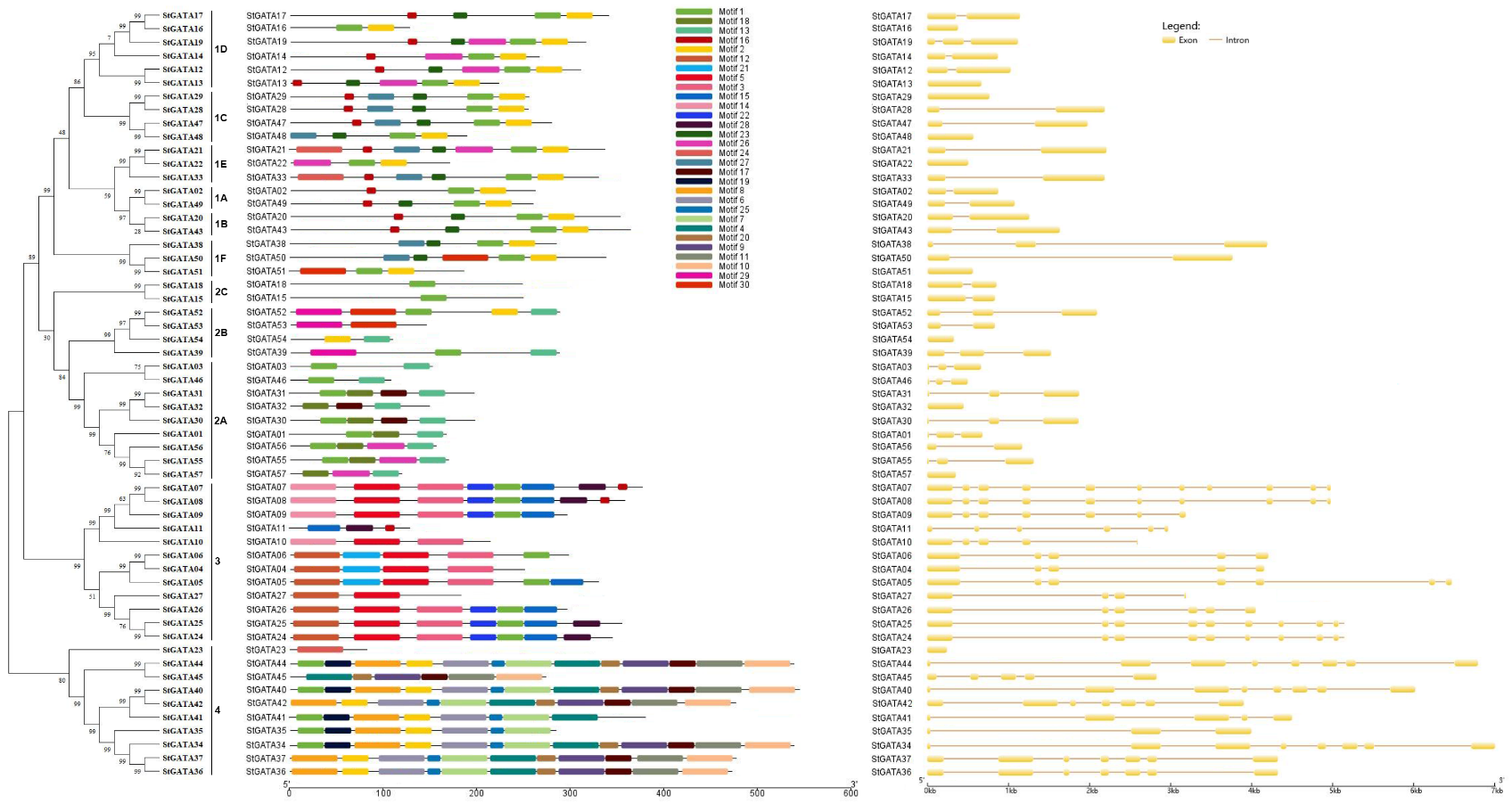


**Supplementary Figure S2.** Phylogenetic relationship, motif composition and gene structure of StGATA gene members. (A) The phylogenetic tree was constructed based on the neighbor-joining method with 1,000 bootstrap replicates. (B) Motif composition of StGATA proteins was analyzed using MEME software; The motifs, numbered 1-20, are suggested in different colored boxes; Protein length is evaluated using the scale at the bottom. (C) Exon/intron distribution of StGATA genes was performed using GSDS 2.0; The yellow boxes indicate exons; The blue boxes indicate untranslated region (UTR); The lines suggest introns; Gene length is evaluated using the scale at the bottom.

**
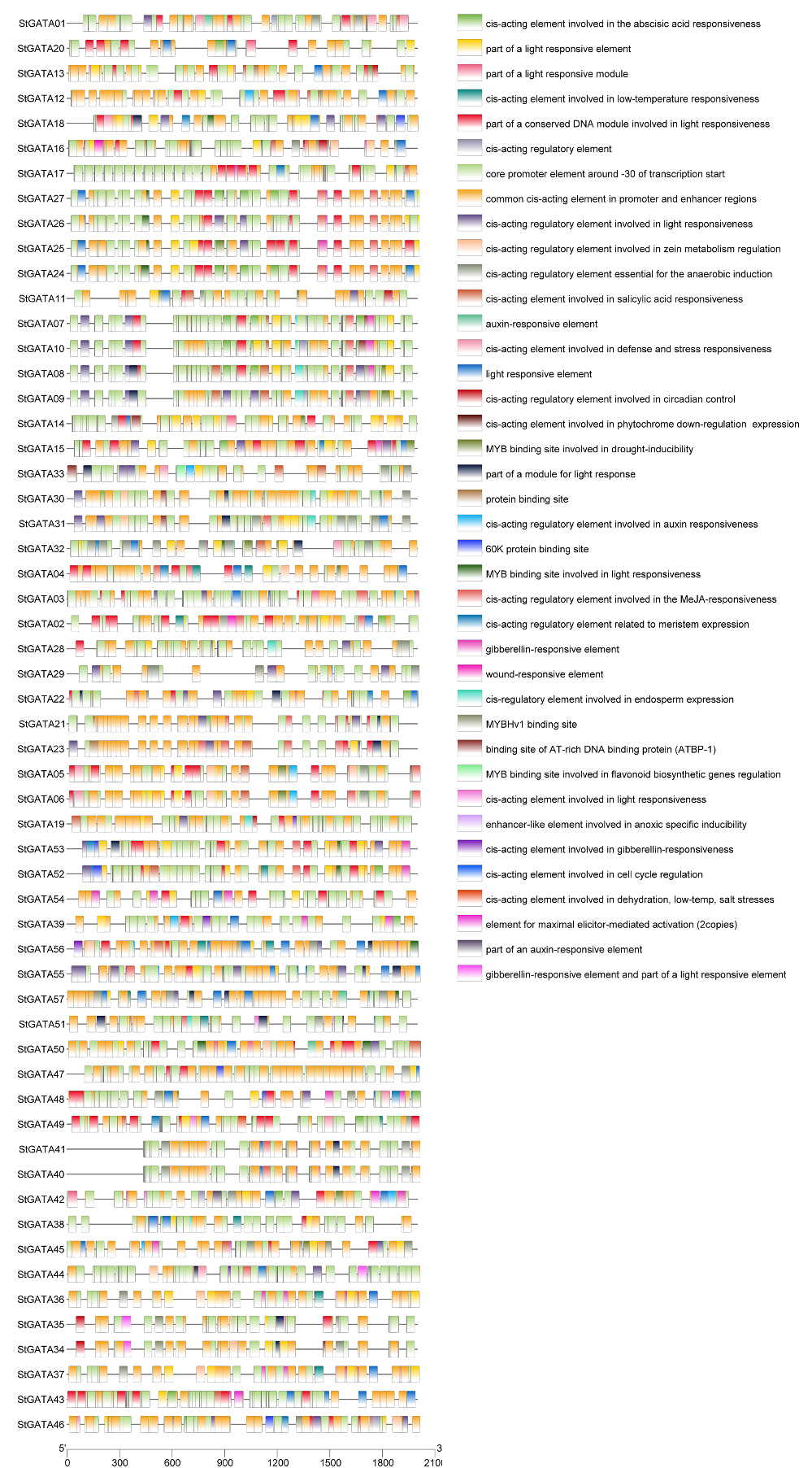
**

**Supplementary Figure S3.** Cis-regulatory elements in the promoter regions of StGATA genes in potato. The length of promoter regions can be estimated using the scale at the bottom.


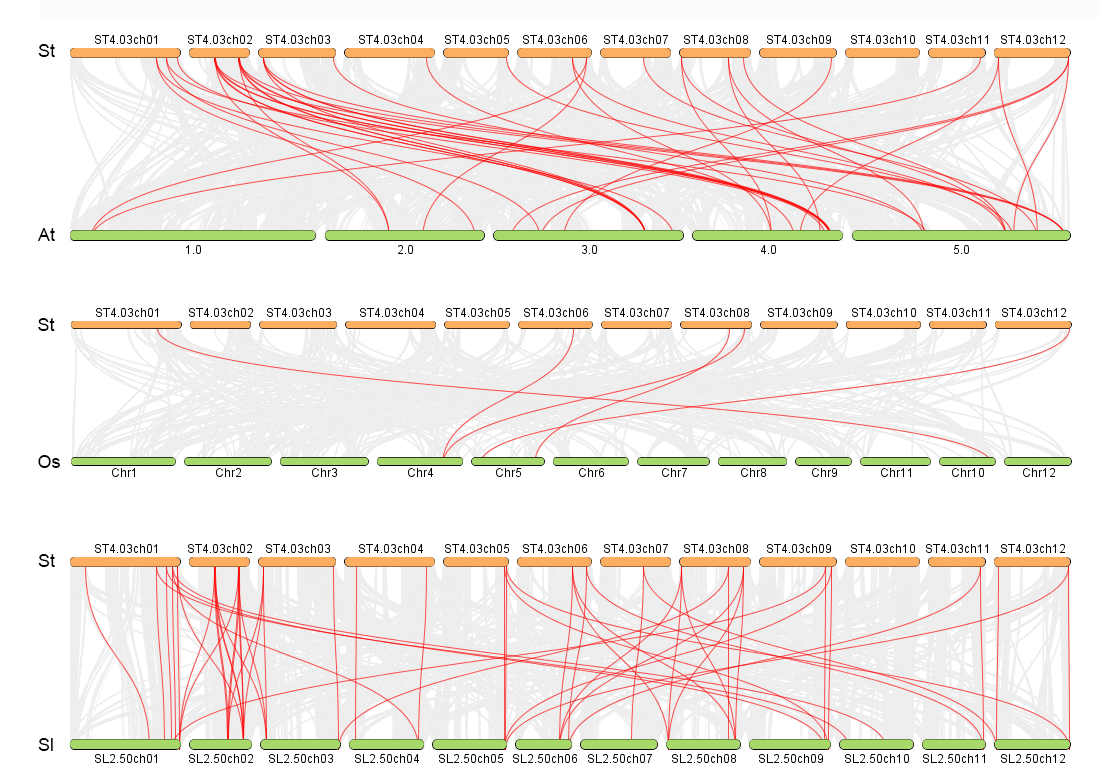


**Supplementary Figure S4**. Synteny analysis of GATA genes between *Solanum tuberosum* (St) and *Arabidopsis thaliana* (At), *Oryza sativa* (Os), or *Solanum lycopersicum* (Sl). MCScanX software was used to analyze the syntenic relationship. Red lines highlight the syntenic GATA gene pairs; Grey lines in the background represent the collinear blocks within potato plants and other plant genomes.

**Supplementary Tables**

**Supplementary Table S1** The gene information of GATA gene family identified in potato plants.

**Supplementary Table S2** Ka, Ks, Ka/Ks, and divergence time (MYA).

**Supplementary Table S3** Conserved Motifs in *StGATA* family members in potato.
