# Supplementary material for: Genome-Wide Identification of GATA Family Genes in Potato and Characterization of StGATA12 in Response to Salinity and Osmotic Stress
Source: Int J Mol Sci. 2024 Nov 19;25(22):12423. doi: 10.3390/ijms252212423 (PMC11594768; doi:10.3390/ijms252212423)
Supplement: Supplementary file 1 [file ijms-25-12423-s001.zip › Supplementary Materials.pdf]

Supplementary Figures

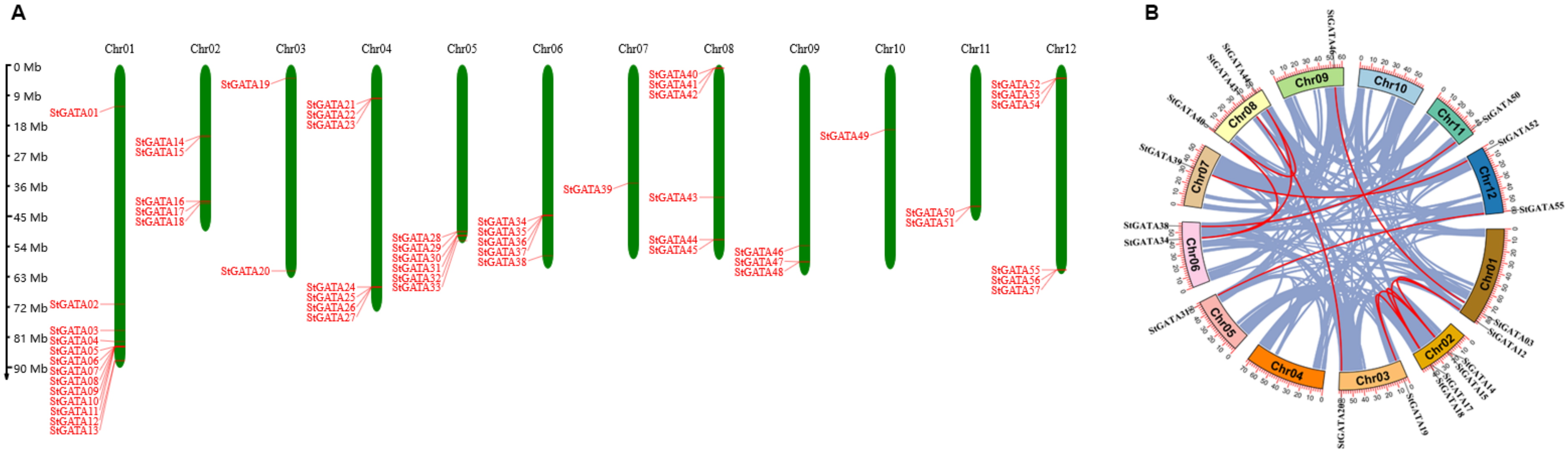

Supplementary Figure S1. Chromosomal localization, segmental duplication, and phylogenetic tree of StGATA proteins. (A) Chromosomal localization of *StGATA* genes in potato plants. (B) Schematic representations of inter-chromosomal relationships of *StGATAs*. Gene duplication events were analyzed by MCScanX. Blue lines inside present all synteny blocks in the potato genome. The segmentally duplicated genes were linked by the bold red line.

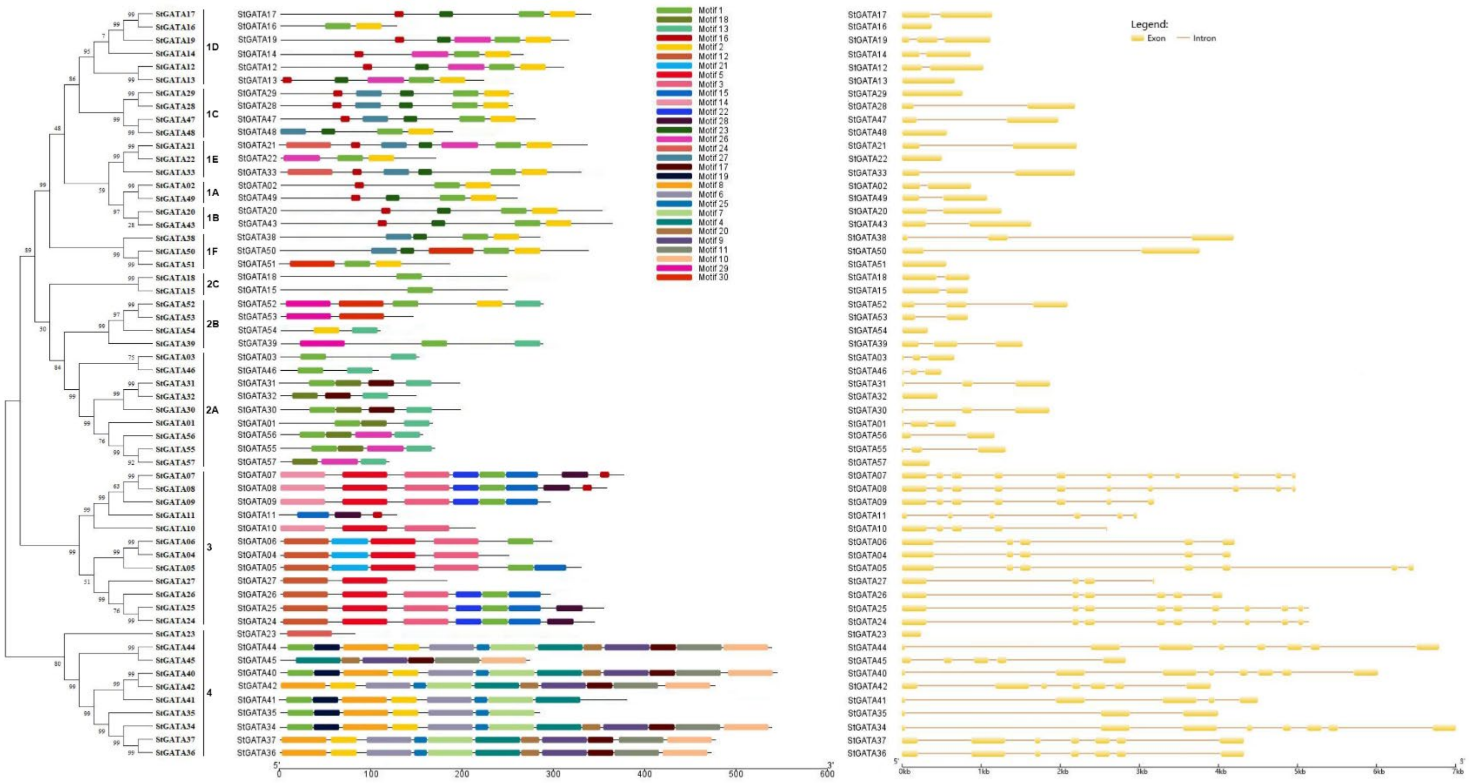

Supplementary Figure S2. Phylogenetic relationship, motif composition and gene structure of StGATA gene members. (A) The phylogenetic tree was constructed based on the neighbor-joining method with 1,000 bootstrap replicates. (B) Motif composition of StGATA proteins was analyzed using MEME software; The motifs, numbered 1-20, are suggested in different colored boxes; Protein length is evaluated using the scale at the bottom. (C) Exon/intron distribution of StGATA genes was performed using GSDS 2.0; The yellow boxes indicate exons; The blue boxes indicate untranslated region (UTR); The lines suggest introns; Gene length is evaluated using the scale at the bottom.

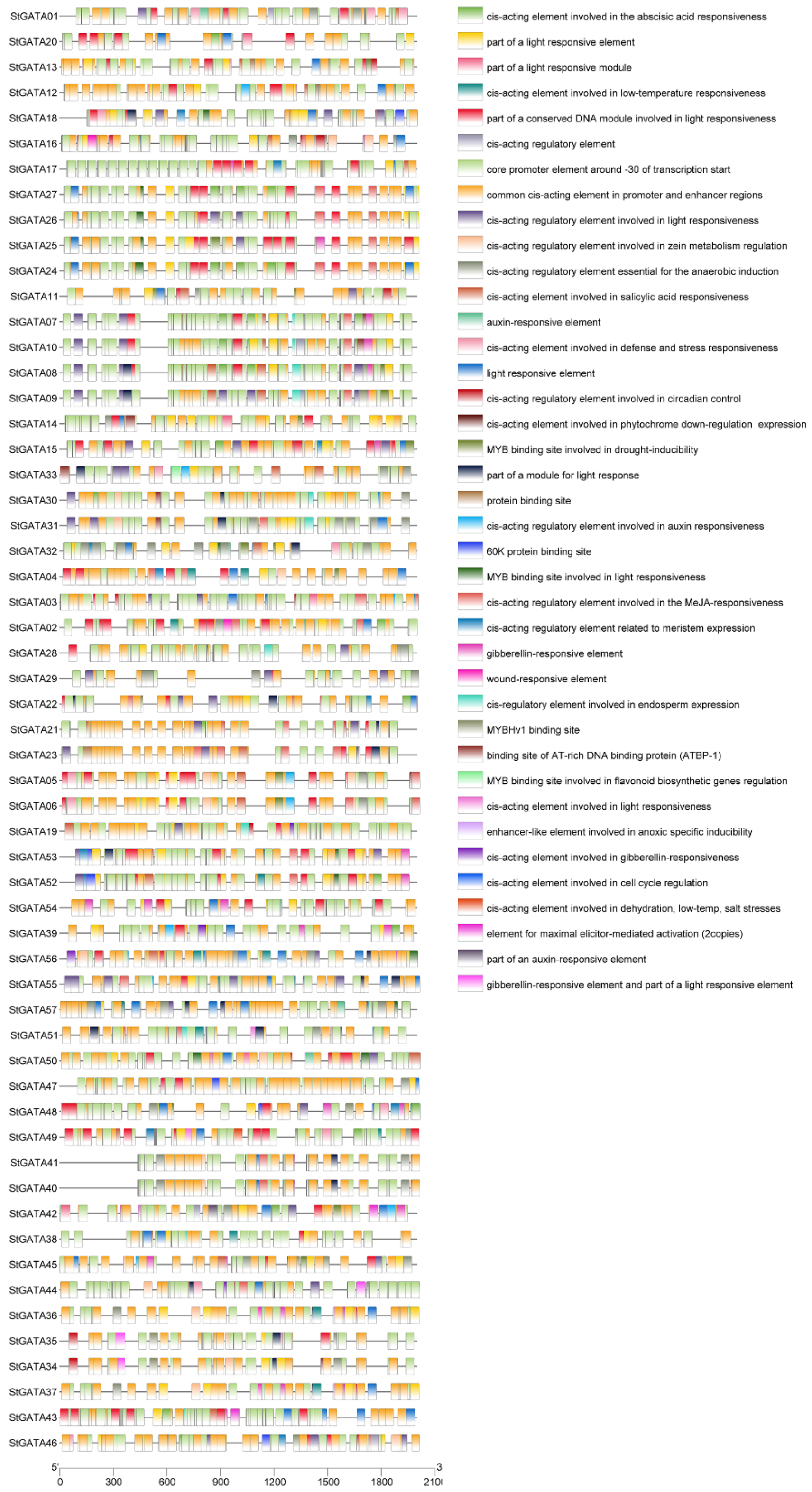

Supplementary Figure S3. Cis-regulatory elements in the promoter regions of StGATA genes in potato. The length of promoter regions can be estimated using the scale at the bottom.

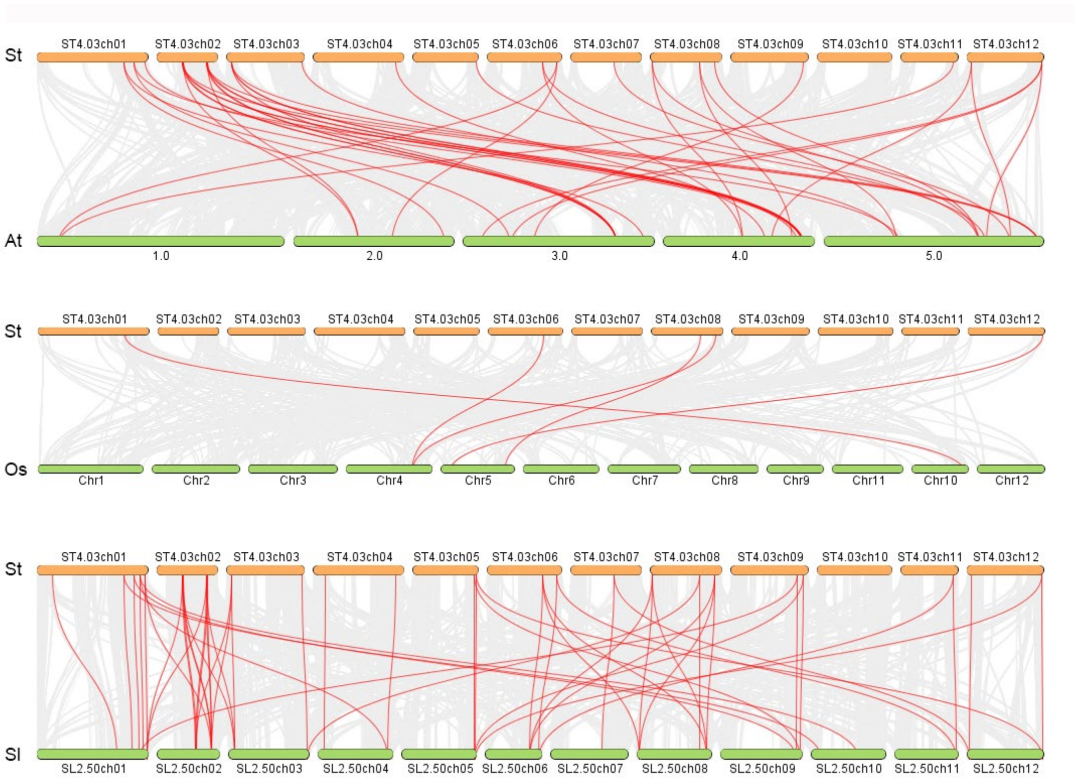

Supplementary Figure S4. Synteny analysis of GATA genes between *Solanum tuberosum* (St) and *Arabidopsis thaliana* (At), *Oryza sativa* (Os), or *Solanum lycopersicum* (Sl). MCScanX software was used to analyze the syntenic relationship. Red lines highlight the syntenic GATA gene pairs; Grey lines in the background represent the collinear blocks within potato plants and other plant genomes.

Supplementary Tables

Supplementary Table S1 The gene information of GATA gene family identified in potato plants.

Supplementary Table S2 Ka, Ks, Ka/Ks, and divergence time (MYA).

Supplementary Table S3 Conserved Motifs in *StGATA* family members in potato.
