# Supplementary material for: Genome-Wide Identification of GATA Family Genes in Potato and Characterization of StGATA12 in Response to Salinity and Osmotic Stress
Source: Int J Mol Sci. 2024 Nov 19;25(22):12423. doi: 10.3390/ijms252212423 (PMC11594768; doi:10.3390/ijms252212423)
Supplement: Supplementary file 1 [file ijms-25-12423-s001.zip › Supplementary Table S3.pdf]

Additional Table S3 Conserved Motifs in *StGATA* family members in potato.

motif1	HCGVTKTPQWRAGPLGPKTLCNACGVRYK
motif2	PEYRPAASPTFSGPAHSNSHRKVLEMRKK
motif3	DPTRQNISPRMASLIRFREKRKERCFDKKIRYAVRKEVAQRMHRKNGQFA
motif4	DHLEKLQILGHHNSPLCHIDLKDVLNYEEFTSHLSSDEQQQLLKYLPPVD
motif5	AGPVDGGGGMAEPSRTSELTLSFEGEVYVFPAVTPEKVQAVLLLLGGCEV
motif6	VEKLTKDLCTILHEQQSSYFSGSSEEDLLFESDKPMVSVEIGHGSVLIRH
motif7	DNKHRSVNEAYSRLSTPPVNINKGVNLPNLGTERTKKPNGQGMEQHQIKR
motif8	LKNKEAKVLKQKQNHDNTVVRTPPDYYQGFLKGLDEDTSNRSSSGSAISN
motif9	FQKLLAEGVFDNSLSGVTIEDCRNLKRFILCYLTKSKWVEQYNLLKDTKC
motif10	FRSFNEASDQDLLLDVPSNSSFPQAELLLPTSSFAAQASTSSSSVYPHLV
motif11	GQHPKYSGAKTTMKSPKRVVMKSSYEQKELVDNDSSCFSPKSLFALPSEN
motif12	NPRPLQARPYEDHAPLPSIRMVEEEEEEEDDDDGAYEDDGGEETMDEAED
motif13	RSLSTHRRKLGEEEQAAFLLMALSCGSVY
motif14	MEAADNSRNMLGGQYGVVAFQQEYMNVPIRVDRYGGGGFEADDISVGGGY
motif15	NKGMLRDITKGGSHVPFDKNEPGTPDIKLSTFAPEN
motif16	ELEWLSNFVED
motif17	KSSSSGSEVAGGPNVVGTGHSVNVKKPLE
motif18	KKRRALLGLNKDDKKSKKSLAKSHKNHEA
motif19	TKGTLVNYTPLHARAEPDDLEEYRVSRFK
motif20	SFAPPDSLRSMFESSQFEENL
motif21	MDNPTPHIRYDQHHHSHSHALHNGGAGGSMEMNGVEGVSHN
motif22	EGECDSADNIDEGDGAAHSEPTLGKCHHC
motif23	PVKARSKRRRPAARNW
motif24	IDCSSFFDHMEDLIEFSPENECGGLDAVDCKDFPSIWNDPLPDSDPLFSG
motif25	SIGREEESEASSLSV
motif26	SSNSPSSFSSSGVSTNESIVKNECPPYKKRKLHFSREVGLQ
motif27	GIFDHHSPVSVLENSSSCSGSSSNCNVNG

motif28	HEEDMQEATENFADSSPFCIGSSSVNIDEE
motif29	YNNNYQFSSSSTNSSCQTFFNISTTTNIQDQSGYDYHSHQFHQPQHQHEV
motif30	GIHDALEKKKEKGLKLSLCKGAEEKMKNLKLEDNDAAIIESDVSKKKISNN
